# Editorial: Glucocorticoid and bone: Friend or foe

**DOI:** 10.3389/fendo.2022.995124

**Published:** 2022-08-11

**Authors:** Ulrike Baschant, Barbara Hauser, Elizabeth Martha Winter

**Affiliations:** ^1^ Division of Endocrinology, Diabetes and Bone Diseases, Department of Medicine III and Center for Healthy Aging, Technische Universität Dresden, Dresden, Germany; ^2^ Centre for Genomic and Experimental Medicine, Institute of Genetics and Cancer, University of Edinburgh, Edinburgh, United Kingdom; ^3^ Rheumatic Diseases Unit, Western General Hospital, Edingburgh, United Kingdom; ^4^ Department of Internal Medicine, Division of Endocrinology, Center for Bone Quality, Leiden University Medical Center, Leiden, Netherlands

**Keywords:** glucocorticoid, bone, osteoporosis, treatment, secondary osteoporosis

## Introduction

**Figure 1:**
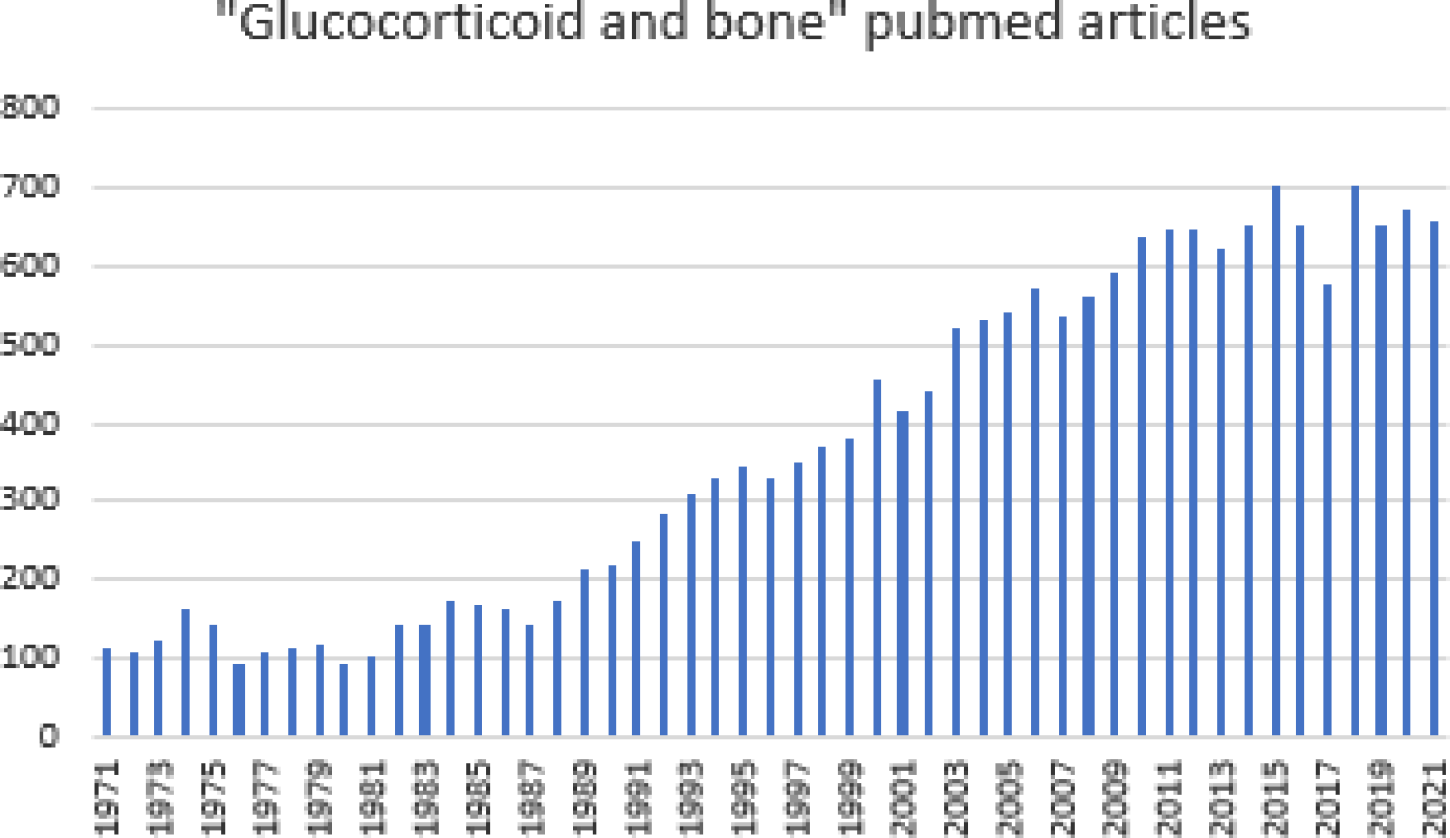
Number of publications listed in pubmed on "glucocorticoids and bone" over the last 50 years.

Glucocorticoids have potent anti−inflammatory effects and their discovery in 1948 improved therapy for many diseases with chronic inflammation noticeably. However, glucocorticoid treatment causes severe side effects, including bone loss and increased fracture risk. The first studies on the interaction between glucocorticoids and bone were published shortly after their discovery over 60 years ago and interest on the interaction between endogenous and exogenous glucocorticoids and bone grows (**Figure 1**). The collection of articles on the topic “Glucocorticoid and bone: friend or foe” consists of original and review articles which summarize and elaborate on current knowledge of basic mechanisms of glucocorticoid hormones and their receptors in bone cells and on the clinical aspects of treatment and prevention of glucocorticoid-induced osteoporosis (GIOP).

## Exogenous and endogenous glucocorticoid metabolism and bone

Endogenous glucocorticoid hormones are main mediators of stress responses and besides regulating immune responses, they influence whole body homeostasis, metabolism and tissue homeostasis including the skeletal system. The activity of glucocorticoids within the cells is controlled by the enzymes 11β-hydroxysteroid dehydrogenases 1 and 2 (11β-HSD1, 11β-HSD2) acting in opposing manners. 11β-HSD1 converts the inactive hormone cortisone into the active form cortisol, whereas 11β-HSD2 oxidizes cortisol into cortisone. Local pre-receptor metabolism of glucocorticoids by 11ß-HSDs contributes to cell-type and tissue specific actions of glucocorticoids that may influence metabolism and also bone homeostasis. Martin et al. review the role of 11ß-HSD enzymes and local metabolism in bone homeostasis and bone function and discuss strategies how to modulate local glucocorticoid metabolism in order to treat bone diseases.

Activated glucocorticoids bind to the glucocorticoid receptor, a nuclear receptor that induces transactivation or transrepression of different target genes depending on cell types. Lee et al. discuss in their review the complexity of glucocorticoid actions in different bone cell types on a molecular level. They further summarize recent studies on influences of therapeutic glucocorticoids on circadian rhythm of endogenous glucocorticoid levels and the consequent impact of the disturbed circadian rhythm on bone integrity.

The article of Gado et al. summarizes the effects and molecular mechanisms of therapeutic glucocorticoids on bone cells, specifically on osteoblasts and osteocytes and highlight their implications for clinical therapy of GIOP.

The clinical impacts of endogenous hypercortisolism on phosphate homeostasis are investigated by Bosman et al. in a retrospective study on 99 patients with Cushing’s syndrome (CS). 16% of patients with CS had hypophosphatemia, which was associated with increased cortisol urinary excretion. In a subset of patients, serum phosphate level increased significantly after CS patients went into remission. The authors postulate that possible mechanisms for urinary phosphate excretion could include FGF23, BMI and parathyroid hormone levels.

## Treatment and prevention of glucocorticoid-induced osteoporosis

Hayes et al. addresses the difficulties and uncertainties on starting and stopping bone-protective medication in GIOP. They state that there is a low awareness of GIOP but also a lack of clear guideline recommendations in particular for when to stop osteoporosis treatment. Based on current evidence the advice is to stop bone-protective medication 6-12 months after glucocorticoid discontinuation, since fracture remains elevated for about one year following glucocorticoid treatment cessation. Since it is widely known from the Denosumab and Teriparatide Administration (DATA) extension study that teriparatide followed by denosumab is effective for treatment-naïve postmenopausal osteoporotic women with an increase in femoral neck, total hip and spine BMD (1), it is interesting to see whether this also counts for patients on glucocorticoids. Hirooka et al. investigated sequential treatment strategies in GIOP patients who were pre-treated with bisphosphonates. The study demonstrates that the treatment sequence of two years of teriparatide followed by two years of denosumab leads to higher femoral neck bone mineral density (BMD) gain than with 4 years of continuous denosumab treatment (non-randomized). Only little is described on herbal medicines for GIOP. Zhang et al. review the potential use of herbal compounds. They describe that compounds like escin, ginsenosides and glycyrrhizic acid exert anti-inflammatory properties like glucocorticoids, but without inducing GIOP, and also compounds such as tanshinol and icariin that alleviate GIOP through mechanisms including regulation of Wnt and RANKL/RANK signaling.

## GIOP in various diseases

This section includes articles on bone health and fracture risk of rare conditions, which are frequently treated with high dose glucocorticoids.

Box et al. review current evidence and mechanism of bone loss and increased fracture risk in large and small vessel vasculitides with a particular focus on the impact of high dose glucocorticoids on bone health. The article also elaborates on other factors that increase fracture risk including chronic inflammation, organ involvement such as chronic kidney disease and relative immobility. The increasing use of adjunctive glucocorticoid-sparing treatments may have a potential positive impact on fracture risk in patients with vasculitis.

The observational study by Liu et al. presents quantitative computer tomography BMD data of nineteen patients with Duchenne muscular dystrophy (DMD) treated with high dose glucocorticoids. The study shows a gradual overall BMD loss over 2 years at the lumbar spine. A multilevel mixed effect model identified age and functional activity scores but not cumulative glucocorticoid exposure as independent predictors of BMD loss.

Rymuza et al. describes the impact of intravenous methylprednisolone on bone microarchitecture in 15 patients with graves orbitopathy. The study shows that trabecular bone score decreased significantly in 33% of patients treated with high dose intravenous methylprednisolone. The authors highlight the need for fracture risk and BMD assessment in these patients.

In summary, glucocorticoid effects on bone are still not completely understood, thus this topic is still a major research focus. The Research Topic provides state-of-the-art reviews and novel molecular and therapeutic insights into the dichotomous relationship between glucocorticoids and bone. Overall, novel insights into the pathogenesis of GIOP may provide better prevention and treatment strategies of affected patients.

## Author contributions

All authors listed have made a substantial, direct, and intellectual contribution to the work and approved it for publication.

## Conflict of interest

The authors declare that the research was conducted in the absence of any commercial or financial relationships that could be construed as a potential conflict of interest.

## Publisher’s note

All claims expressed in this article are solely those of the authors and do not necessarily represent those of their affiliated organizations, or those of the publisher, the editors and the reviewers. Any product that may be evaluated in this article, or claim that may be made by its manufacturer, is not guaranteed or endorsed by the publisher.
